# Results of the Last 5 Years (2018-2022) of the Specialist Title Exam of The Brazilian College of Surgeons

**DOI:** 10.1590/0100-6991e-20243749-en

**Published:** 2024-04-22

**Authors:** GERSON ALVES PEREIRA, PEDRO TADAO HAMAMOTO-FILHO, ROBERTO RASSLAN, DYEGO SÁ BENEVENUTO, EDUARDO NACUR SILVA, ALEXANDRE FERREIRA OLIVEIRA, PEDRO EDER PORTARI

**Affiliations:** 1 - Universidade de São Paulo (USP), Curso de Medicina - Bauru - SP - Brasil; 2 - Universidade do Estado de São Paulo (UNESP), Faculdade de Medicina de Botucatu - Botucatu - SP - Brasil; 3 - Hospital das Clínicas da FMUSP, Divisão de Clínica Cirúrgica III - São Paulo - SP - Brasil; 4 - Hospital Copa Star, Cirurgia do Aparelho Digestivo - Rio de Janeiro - RJ - Brasil; 5 - Santa Casa de Belo Horizonte, III Clínica Cirúrgica - Belo Horizonte - MG - Brasil; 6 - Faculdade de Medicina da Universidade Federal de Juiz de Fora, Oncologia - Juiz de Fora - MG - Brasil; 7 - Universidade Federal do Estado do Rio de Janeiro (UNIRIO) Escola de Medicina e Cirurgia - Rio de Janeiro - RJ - Brasil; 8 - Presidente do Colégio Brasileiro de Cirurgiões - Rio de Janeiro - RJ - Brasil

**Keywords:** Employee Performance Appraisal, General Surgery, Simulation Training, Avaliação de Desempenho Profissional, Cirurgia Geral, Treinamento por Simulação, Sociedades Médicas

## Abstract

The article discusses the evolution of the Brazilian College of Surgeons (CBC) specialist title exam, highlighting the importance of evaluating not only theoretical knowledge, but also the practical skills and ethical behavior of candidates. The test was instituted in 1971, initially with only the written phase, and later included the oral practical test, starting with the 13th edition in 1988. In 2022, the assessment process was improved by including the use of simulated stations in the practical test, with the aim of assessing practical and communication skills, as well as clinical reasoning, in order to guarantee excellence in the assessment of surgeons training. The aim of this study is to demonstrate the performance of candidates in the last five years of the Specialist Title Test and to compare the performance results between the different surgical training groups of the candidates. The results obtained by candidates from the various categories enrolled in the test in the 2018 to 2022 editions were analyzed. There was a clear and statistically significant difference between doctors who had completed three years of residency recognized by the Ministry of Education in relation to the other categories of candidates for the Specialist Title..

## RESUMO

O artigo aborda a evolução da prova de título de especialista do Colégio Brasileiro de Cirurgiões (CBC), destacando a importância de avaliar não apenas o conhecimento teórico, mas também as habilidades práticas e o comportamento ético dos candidatos. A prova foi instituída em 1971, inicialmente com apenas a fase escrita, e posteriormente foi incluída a prova prática oral, a partir da 13ª edição em 1988. Em 2022, foi aprimorado o processo de avaliação, incluindo na prova prática o uso de estações simuladas, visando avaliar habilidades práticas e de comunicação, além do raciocínio clínico, buscando garantir a excelência da avaliação da formação dos cirurgiões. O objetivo deste estudo é demonstrar o desempenho dos candidatos nos últimos cinco anos da Prova de Título de Especialista e comparar os resultados dos desempenhos entre os diferentes grupos de formação cirúrgica dos candidatos. Foram analisados os resultados obtidos pelos candidatos das diversas categorias inscritas na prova nas edições de 2018 a 2022. Ficou evidente e estatisticamente significativa a diferença entre os médicos que fizeram três anos de residência reconhecida pelo MEC em relação aos demais categorias de candidatos ao Título de Especialista.

## INTRODUCTION

The Brazilian College of Surgeons (CBC), founded in 1929, began the selection process for its Title of Specialist in 1971, with only the written phase with multiple-choice questions, validated by RESOLUTION No. 804/1977 of the Brazilian Federal Medical Board (CFM). From its 13th edition, held in 1988, the oral test was included in the evaluation process as a second phase for candidates approved in the written test. 

The public notices of the competitions have been regulated by the Brazilian Medical Association (AMB) since 1991, through an agreement between CBC, AMB, CFM, and the National Commission of Medical Residency (CNRM) of the Ministry of Education (MEC). The CBC’s Specialist Title Commission (COTECIG) has been improving its evaluation process every year. In the first (written) phase, specific questions are requested from invited professors, while in the second (oral) phase, clinical cases use imaging exams to interpret findings and intraoperative situations for diagnostic and therapeutic decision-making. 

Another important step was taken in the evaluation process of candidates for the CBC Specialist Title in the 2022 edition, with the insertion of a practical test using simulated stations in the second phase, complementing the oral test. 

The objective of the Specialist Title test is to use evaluation methods that measure, with acceptable accuracy, the skills necessary for a safe and qualified practice of General Surgery and to identify the suitable candidates who should be approved with satisfactory performance in well-defined skills that comprise all these dimensions. Such competencies include theoretical knowledge, psychomotor skills, interpersonal communication, professional ethics, and clinical reasoning applied to medical practice situations (Epstein and Hundert, 2002), with a focus on General Surgery. 

The notion of competency-based education was associated with the assessment of behavioral goals. For the learning objectives that need to be evaluated, the concept of performance was adopted, i.e., the actions effectively manifested, in which the student should have a given performance, defining the level considered acceptable (Gontijo et al., 2013). The best way to evaluate performance is to define the task to be performed in each context and verify the students’ ability to appropriately apply their competences to perform the task in that situation (Aguiar & Ribeiro, 2010). Thus, the choice of evaluation methods requires clarity of objectives, as well as knowledge of their psychometric properties to identify which ones should be used at each moment (Wass et al., 2007). 

**Figure 1:**
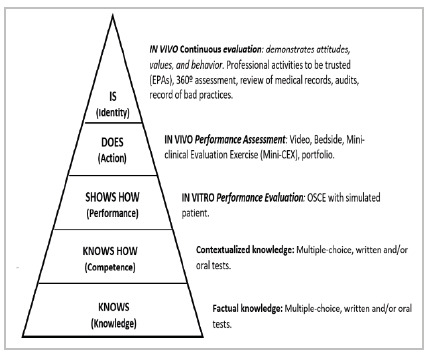
Updated version of Miller’s Pyramid (1990), with examples of what and what forms of assessment should be used at each level. Adapted from Cruess et al., 2016.

The implementation of the competency-based model (Epstein & Hundert, 2002) implies measures by evaluation methods (Norcini et al., 2013). A widely used approach to facilitate such measures was proposed by Miller, who grouped similar competencies into broader domains. Figure 1 shows the updated version of Miller’s Pyramid (Miller, 1990; Boursicot et al., 2011; and Cruess et al., 2016), with examples of what and what forms of assessment should be used at each level.

“Knows” and “knows how” are domains of cognitive competence (Norcini et al., 2013), the professional’s theoretical knowledge. The following domains comprise the competencies related to the practical application of the acquired knowledge. “Shows how” indicates what the professionals can do - their clinical skills (Boursicot et al., 2011). This includes psychomotor skills (physical examination maneuvers, medical procedures, etc.) and behavioral skills (communicating with patients and colleagues, complying with patient safety procedures, etc.). On the other hand, “Does” describes the real physician’s performance: how one really performs one’s functions amid the pressures of a dynamic environment, with its uncertainties and subjectivities, and one’s ethical behavior in the face of emotionally complex situations and flexibility in the face of diverse demands (Bica & Kornis, 2020). 

Cognitive domains are best assessed by written tests, while clinical skills (psychomotor and behavioral skills) are ideally assessed by simulation methods, such as the Objective Structured Clinical Examination - OSCE (Bica & Kornis, 2020). As competencies are specific, proficiency in one does not predict performance in the others (Van Der Vleuten et al., 2010). Thus, only a combination of methods assesses a student’s overall aptitude, as no single method is effective for all three domains: cognitive, psychomotor, and behavioral.

If the Specialist Title test had only written questions, its validity would be compromised, as it would exclude all practical skills from the evaluation. Thus, the definition of a cognitive (theoretical) test as an assessment of “knowledge, skills, and attitudes” is meaningless. To approximate these objectives, assessments should, at a minimum, include simulation scenarios (such as OSCEs) to assess clinical skills. Of course, this design is more expensive and laborious, requiring abundant inputs and human resources, which demands a logistical study on the evaluations’ feasibility. However, considering the relevance of the objectives set, this greater investment is essential, as it is not possible to broadly assess the aptitude of candidates with inferior technical quality resources. 

In this scenario, COTECIG has improved the content matrix of the CBC Specialist Title test and intends to expand the training of staff to be reviewers and elaborators of questions for the various tests. Thus, the psychometric properties of each type of test described in Table 1 have been considered.

**Table 1:** Psychometric properties and their definitions used by COTECIG.

Psychometric property	Definition
Reliability	Indicates whether the result of the test allows inferences about the candidates' proficiency in the domain being evaluated (Shumway & Harden, 2003) and for this a good sampling of the content in question and a well-standardized correction are essential.
Validity	Ability to effectively assess what "should" be assessed; also, describes the power of the test to identify the varying levels of candidates’ proficiency.
Trust	Related to the precision, accuracy, objectivity, and reproducibility of the assessment instruments used, components that determine the reliability and consistency of the results obtained.
Acceptability	Indicates whether different actors (candidates, evaluators, and managers) agree on the format and content of the evaluation.
Feasibility/Viability	Considers the varied requirements (human and material resources, space conditions, time, support material, with adequate planning, convenient organization, and appropriate cost control) necessary to implement a test (Shumway & Harden, 2003).
Educational impact	Relates to the consequences the test produces on the learning process (Wass et al, 2007).

With the clinical simulation, multiple competencies can be tested simultaneously: patient care, medical knowledge, psychomotor skills for procedures, professionalism, and interpersonal skills, among others, allowing both the development and evaluation of individual competencies in similar daily situations and the effective collaboration in teams and the construction of a safety-oriented culture (Pereira Jr, 2021).

The objective of this study is to demonstrate the performance of the candidates in the last five years of the Specialist Title Test and to compare the results of the performances between the different groups of surgical training candidates.

## METHODS

In the 2018 to 2022 editions of the CBC Specialist Title test, two phases were held: the first phase, with 100 multiple-choice questions, and the second one, with an oral test (2018 to 2021) and oral and simulated tests in the 2022 edition. All questions and clinical cases used were elaborated based on clinical practice, aiming at problems demanding the application of principles or solutions that require a complex mental process in General Surgery.

According to the CBC’s notices, candidates could apply in five categories:

- A - Have completed the three years of Medical Residency in General Surgery recognized by CNRM/MEC.
- B - Have completed the Medical Residency in a Basic Surgical Area (two years) recognized by the CNRM/MEC, plus proof of practice in the specialty of General Surgery, through a statement signed by the Director of the Hospital and/or Head of the Surgery Service, in the period between 2018 and 2022.
- C - Have the certificate of completion of the two-year Training in General Surgery issued by the CBC (CBC General Surgery Training Program) plus proof of two years of practice in the specialty of General Surgery, through a statement signed by the Director of the Hospital and/or Head of the Surgery Service.
- D - Have the certificate of completion of the three-year Training in General Surgery issued by the CBC (CBC General Surgery Training Program).
- E - Be registered with the Regional Council of Medicine for at least six years and proof of six years of experience as a general surgeon in Brazil, through a statement signed by the Director of the Hospital and/or Head of the Surgery Service. The proof can be made through documents issued by various Services and Hospitals that, together, reach the minimum of six years required.

In the first phase test, the focus of the evaluation was cognitive knowledge through questions that cover all areas of broad knowledge that compose the matrix of contents of General Surgery. The questions were elaborated and selected by COTECIG members and invited collaborators. 

The test was applied by a specialized company to all candidates at the same time and with the same rules. In the 2018 and 2019 editions, the written test of the first phase was face-to-face, applied in the cities of Belém, Belo Horizonte, Brasília, Cuiabá, Curitiba, Fortaleza, João Pessoa, Maceió, Manaus, Palmas, Porto Alegre, Recife, Rio de Janeiro, Salvador, São Luís, São Paulo, Teresina, and Vitória, consisting of 100 multiple-choice questions composed of four alternatives, one of which is correct, on the topics contained in the exam syllabus. The duration was three hours, with no extension. In the 2020 to 2022 editions, the test was carried out remotely by the same application company. The test corrections and data analysis were carried out by a specialized company, to guarantee the process confidentiality. The candidate who obtained a grade of at least 70% correct answers in relation to the highest grade measured and whose absolute score in the test had at least 50% correct answers was considered approved, thus qualifying for the Oral Practical Test.

In the oral test of the second phase, each candidate individually faced three boards with two or more face-to-face evaluators discussing different standardized clinical cases, with a total duration of 90 minutes (30 minutes in each board), who filled out a standardized evaluation instrument, whose average approval should be equal to or greater than 70%.

In simulated stations test, which complemented the oral practical test in the 2022 edition, candidates were informed by email that this test would have four stations, being advised that each station would have different tasks to ascertain the skills that need to be demonstrated to obtain the CBC Specialist Title, with a variable duration of time (10 to 20 minutes), according to the evaluation objectives. 

The first simulated station concerned surgical technique, the second pertained to care of a surgical clinical case with a simulated patient, and the third contemplated videosurgery. The fourth simulated station would be online, and the digital platform was made available so that everyone could previously know the model of the two simulated stations online to know how it works and how to behave during this type of evaluation. 

During the registration on the digital platform, both the candidates and the evaluators signed the Free and Informed Consent Form (ICF) about the use of data from the computerized checklists of the practical test (oral and simulated) for research approved by the Ethics Committee with CAAE number 60907122.0.0000.5515 and opinion number 5.638.400. 

**Figure 2:**
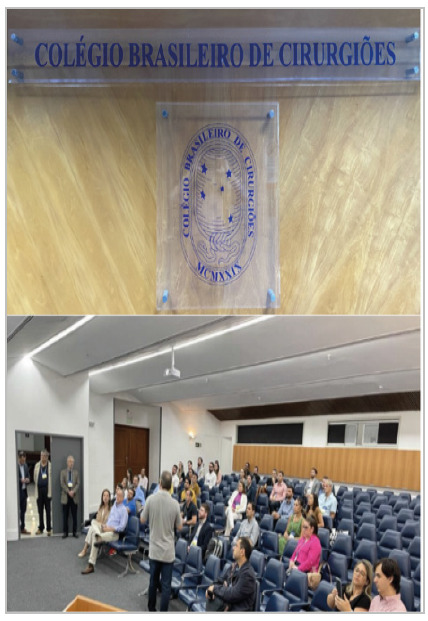
Presentation (pre-briefing) on the required behavior and explanation about the four simulated stations.

Right before the simulated test, with the candidates already identified and without access to the cell phone, a presentation (pre-briefing) was made on the necessary behavior during the simulated stations (Figure 2), explaining that all the necessary information was available: instructions for the simulated scenario, definition of the task(s), execution time of each station, internal evaluator with computerized checklist, and simulated patient (at the Clinical Case Service Station). It was reinforced that in a simulated practical test, it is only possible to evaluate what is verbalized and/or demonstrated by the candidate.

**Figure 3:**
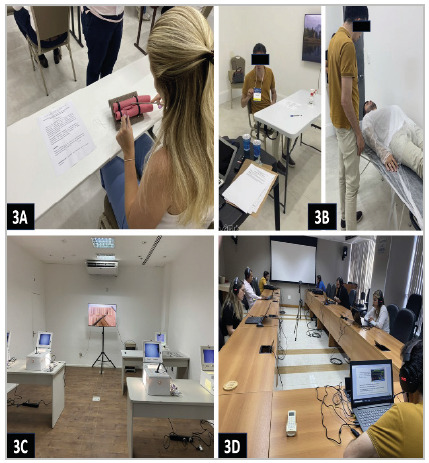
Simulated stations of the practical test of the Specialist Title - CBC Notice 2022. Photos 3A: Manual end-to-side anastomosis station; 3B: Face-to-face simulated station with simulated patient; 3C: Videosurgery station; and 3D: Online simulated station.

The themes of the four simulated stations were also shown:

- STATION 1 - Manual surgical technique - performing a lateral-lateral intestinal anastomosis (duration of 10 minutes) - Figure 3A.
- STATION 2 - Simulated face-to-face case of intestinal obstruction due to rectosigmoid transition tumor, with a simulated patient (duration of 10 minutes) - Figure 3B.
- STATION 3 - Videosurgery technique - performing four black box exercises (duration of 10 minutes) - Figure 3C.
- STATION 4 - Online simulated case of care for severe trauma patient with previously recorded video with simulated nurse who helped the medical candidate (duration of 20 minutes) - Figure 3D.

In the first three simulated stations, the evaluators were face-to-face and in the fourth station, 60 online evaluators (members of the CBC with a specialist title) from all over the country were invited.

All checklists used in the oral and simulated practical tests were computerized, which allowed for a deeper analysis and information cross-referencing. The data were analyzed considering the average performance of the candidates, grouped according to the type of test of the first or second phases. The results of the candidates’ performances in the written tests of the first phase of all editions of the study (2018 to 2022) were joint. The results of the performances of the practical test (oral and simulated) of the second phase were analyzed only in the 2022 edition.

### Statistical analysis

For the analyses, we excluded candidates who were absent from at least one of the exam’s phases. Data distribution was assessed using the Shapiro-Wilk test. Continuous data were compared using the one-way ANOVA test followed by the Bonferroni test. For non-parametric distribution data, we used the Kruskal-Wallis test followed by the Dunn test. Differences between proportions were analyzed using the chi-square test or the Fisher’s exact test. We considered differences with an alpha equal to 5% as statistically significant. The analyses were performed using the SPSS (Statistical Package for the Social Sciences) software, version 21.0 (IBM Corp., Armonk, NY, USA) and the GraphPad Prism software, version 9.5.0 (GraphPad Software, San Diego, CA, USA).

## RESULTS

The number of candidates registered in the CBC Specialist Title exams from 2018 to 2022 were, respectively, 147, 135, 155, 127, and 215, totaling 779 candidates. Fifty candidates were excluded from the examinations due to absence from at least one of the selection phases. Thus, we studied 729 candidates.

We consolidated the results of the candidates’ performances, enrolled in categories A to E in the written tests of the first phase of all editions of the study (2018 to 2022), according to the category of registration, as shown in Table 2. 

**Table 2:** Distribution of candidates by registration category*, average grades, standard deviation, and minimum and maximum grades in the first phase (written test) within the grade from zero to ten, from the 2018 to 2022 editions of the CBC Specialist Title Test.

Category	Number (%)	Average	Standard Deviation	Minim	Maxim
A	187 (25,7)	6.36^†^	0,95	4,00	8,60
B	89 (12,2)	6.56^†^	1,11	1,30	8,70
C	164 (22,4)	5.26^‡^	0,90	2,70	7,70
D	21 (2,9)	5.91^†×^	0,94	4,40	7,60
E	268 (36,8)	5.38^‡×^	1,06	2,00	8,40
Total	729 (100)	5,76	1,13	1,30	8,70

*Categories: A (three years of MEC residency), B (two years of MEC residency - Prerequisite), C (two years of CBC Training), D (three years of CBC Training) and E (six years of documented surgical practice, without medical residency or CBC training). Analysis of variance for all groups is significant for statistical difference (p<0.001). Groups identified by the symbols †‡× are those whose two-by-two comparisons have p>0.05. The absence of similar symbols indicates p<0.05

Table 3 shows the results of failure in the first and second phases, as well as the result of approval in the CBC Specialist Title Test, according to the candidates’ registration category. Comparing the results of the candidates by enrollment category, mainly category A (three years of MEC residency) with category D (three years of CBC training), we see that MEC residents (category A) are approved more than 3 times more (73.8% vs. 23.8%). As for failure, 57.1% of the three-year CBC trainees failed in the first phase (written test) and 19% in the second phase (practical test). In the case of R3 MEC, the failure rate in the first phase is 12.3%, and in the second phase, 13.9%. All these differences were statistically significant. 

**Table 3:** Results of approval/failure of candidates, according to the category of registration* in the 2018 to 2022 notice.

Category	Failed in the first phase (%)	Failed in the second phase (%)	Approved (%)
A	12,3	13,9	73,8
B^†^	27,0	29,2	43,8
C^‡^	53,7	20,7	25,6
D^†‡^	57,1	19,0	23,8
E^‡^	45,5	24,6	29,9

*Categories: A (three years of MEC residency), B (two years of MEC residency - Prerequisite), C (two years of CBC Training), D (three years of CBC Training) and E (six years of documented surgical practice, without medical residency or CBC training). Chi-square analysis within all groups significant for statistical difference (p<0.001). Groups identified by the symbols †‡× are those whose paired comparisons displayed p>0.05. The absence of such symbols indicates p<0.05.

**Figure 4:**
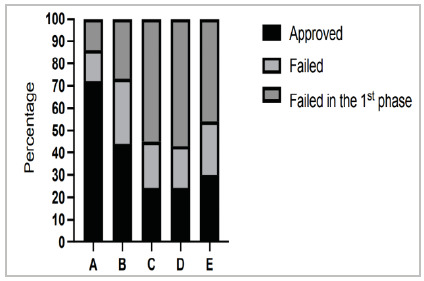
Approval/failure of candidates, according to the category of registration* in the 2018 to 2022 exams. *Categories: A (three years of MEC residency), B (two years of MEC residency - Prerequisite), C (two years of CBC Training), D (three years of CBC Training), and E (six years of documented surgical practice, without medical residency or CBC training). Chi-square analysis within all groups was significant for statistical difference (p<0.001).

Figure 4 shows the results presented in Table 3. Although we no longer have the two-year Basic Area Programs for MEC residents and the two-year Basic Area Programs for CBC trainees, we can observe that the performance of the R2 MEC was superior to the three-year CBC trainees (approval of 43.8% vs. 23.8%), with less than half of the failure in the first phase (27% vs. 57.1%) and greater failure in the second phase (29.2% vs. 19%). 

Another observation is that the performance of the candidates with two and three years of CBC training was quite similar (approval: 25.6% vs. 23.8%; failure in the first phase: 53.7% vs. 57.1%; and failure in the second phase: 20.7% vs. 19%). Although the difference is not statistically significant, category E candidates performed better than two- and three-year CBC trainees, even without formal training, as it should be for MEC Residency and CBC Training.

A the end of the second phase of the 2022 edition of the CBC Title of Specialist in General Surgery exam, on 03/26/2023 (Sunday), the 131 candidates approved in the first phase took the oral test in a hotel and the simulated test on the premises of the CBC building, divided into groups that rotated the tests in the two places, with activities from 8:30 a.m. to 7 p.m.

**Figure 5:**
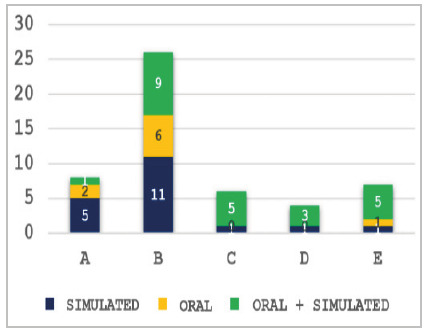
Failure of candidates in the second phase of the 2022 edition, according to the registration category* and the type of practical test. *Categories: A (three years of MEC residency), B (two years of MEC residency - Prerequisite), C (two years of CBC Training), D (three years of CBC Training), and E (six years of documented surgical practice, no medical residency or CBC training).

Figure 5 shows the candidates who failed in the second phase of the 2022 edition, with the insertion of the simulated stations associated with the oral test, divided by registration category. Poor performance in the simulated stations was responsible for most failures, both in isolation and associated with poor performance in the oral practical test.

## DISCUSSION

The first observations about the results concern the number of candidates who annually register for the CBC Specialist Title exam, since there are 454 medical residency programs approved by the National Commission of Medical Residency (CNRM), with 1,598 total vacancies (574 for the first year - R1) for medical residency (Scheffer et al., 2023). By decision of the CNRM, the Basic Surgery Area Prerequisite Program (PPRACB), created in 2019, ceased to exist and was offered as of 2022, but we had 1,074 first year residents in this program in 2021 (Scheffer et al., 2023), which in the three years of offering had three R1 entries and three R2 exits, totaling 3,222 doctors. In addition to these potential candidates, there are also graduates of the 54 Training Centers accredited by the CBC (18 in São Paulo, 17 in Minas Gerais, seven in Rio de Janeiro and Paraná, three in Bahia, one in Espírito Santo, and one in Santa Catarina). The total number of physicians who graduate annually from CBC Training is 150. 

Therefore, there are many potential candidates to the CBC Specialist Title who are not applying for the annual editions. There are many surgeons able to take the exam, in addition to graduates who finish their training annually and could also apply.

Another observation about the candidates of the CBC Specialist Title exam is the large number of applicants in category E, who are physicians registered with the Regional Councils of Medicine for at least six years, and with proof of six years of experience as a general surgeon in Brazil through a statement signed by the Director of the Hospital and/or Head of the Surgery Service. The proof can be made through documents issued by various Services and Hospitals that, together, reach the minimum six years required. This category, representing 36.8% of the candidates in the last five years, although devoid of other titles and therefore more in need of the title granted by the CBC, needs to be rediscussed in the current moment of increasingly specialized professional training demands. New discussions with the CFM and the AMB should be held to align the certification of colleagues without formal training and the needs of public and supplementary health.

The introduction of the practical test with simulated stations served to broaden the focus of the face-to-face assessment of psychomotor skills (manual anastomosis and videosurgery exercises) and communication ones, clinical reasoning, interpretation of complementary exams, and diagnostic and therapeutic decision-making, both in the face-to-face station with a simulated patient (clinical case of intestinal obstruction due to rectosigmoid transition tumor) and in the online simulated care of a severely traumatized patient, in which the simulated nurse asked questions and inquired about the conduct to be taken in each phase of the primary and secondary assessment.

With the computerization of all the checklists used in the oral and simulated practical tests, there was a deepening of the analysis and cross-referencing of information. Thus, for the first time it was possible for COTECIG to compare the results between the candidates’ categories. 

In the results presented, the following performances stand out:

1) category A (three years of MEC residency) versus category D (three years of CBC training) with approval more than three times higher (73.8% vs. 23.8%);
2) the extinct two-year Basic Area Programs for MEC residents, whose performance was superior to the three-year CBC trainees;
3) the similar performance of candidates with two and three years of CBC training; and
4) the better performance of category E candidates in relation to two- and three-year CBC trainees.

From the above analyses, we can infer that:

- the pass rate of CBC trainees is far below that of MEC residents;
- the third year of medical residency conferred greater performance in the approval of MEC residents than of those with only two years, both in the first and second phases, being more than double in both phases;
- the third year of CBC training brought practically no gain in relation to the candidates who had two years of training in the two phases of the test and with lower performance than the candidates in category E.

The sample of graduates of MEC residency programs and CBC trainees is very small and, therefore, we cannot have further inferences, but the results described above are clear and maintained each year in the period of five years of application of the two phases of the Specialist Title. 

Even with the much higher performance of candidates with three years of MEC residency (73.8%), COTECIG considers that this percentage should be much higher if these medical residency programs had a large volume of patients treated and operated, with many opportunities for care and surgical procedures for resident physicians, with trained staff, both from a technical and pedagogical point of view, having professional updating programs with periodic evaluations, as recommended by Resolution No. 2, of May 17, 2006 of the CNRM, which regulates and clarifies the evaluation procedures of resident physicians working in medical residency programs authorized and offered by institutions accredited by MEC. 

With the above analyses, we have considerations about the possible educational impacts that these results of the Specialist Title exam can bring to the Medical Residency and CBC Training Programs, even more so now that the CNRM has published Resolution No. 4, of November 1, 2023, which brings several improvements in the evaluation of resident physicians in relation to the previous Resolution (2006), opening space for the collaboration of the Specialty Societies. 

Thus, in view of these results, the CBC should develop an action plan, together with the MEC Medical Residency Programs and the CBC Training Centers in General Surgery. It would be important for these partnerships to be able to set educational objectives to achieve the necessary competency milestones at each moment of training and, more specifically, at each year of medical residency in General Surgery, through annual updating programs aimed at each of the three years of medical residency, and evaluated following the precepts of the new CNRM resolution (2023). 

Another important action that could be carried out by the CBC is the training of medical residency preceptors in General Surgery. There are several of these training courses for preceptors in various initiatives, but none is specific to the preceptor of Surgery.

## CONCLUSION

Guaranteeing the population of the various regions of the country a safe and qualified medical-surgical practice is a demand that can no longer be ignored by the Brazilian College of Surgeons or by any other Medical Specialty Society. 

COTECIG has sought to improve its techniques for the preparation and review of items, the selection of clinical cases for the oral test, as well as the simulated stations, progressively improving the training of face-to-face and online evaluators. Thus, the commitment to excellence and the constant search for improvement are essential to ensure that obtaining the Title of Specialist by the Brazilian College of Surgeons is recognized as an indication of proficiency and competence in the surgical area.

The difference between physicians who completed three years of residency recognized by MEC in relation to the other categories of candidates for the Title of Specialist was evident and statistically significant.

There are many possibilities of actions to be carried out by the CBC in partnership with the Medical Residency Programs and, mainly, with the Approved Training Centers so that there is a better quality of training for Brazilian surgeons.

## References

[1] Aguiar AC, Ribeiro ECO (2010). The concept and evaluation of skills and competence in medical education current expert perspectives.. Rev. Bras. Educ. Med.

[2] Bica RBS, Kornis GEM (2020). Medical licensing examinations - are they a good idea for medical education in Brazil. Interface (Botucatu).

[3] Boursicot K, Etheridge L, Setna Z, Sturrock A, Ker J, Smee S (2011). Performance in assessment consensus statement and recommendations from the Ottawa conference. Med Teach.

[4] Cruess RL, Cruess SR, Steinert Y (2016). Amending Miller's Pyramid to Include Professional Identity Formation. Acad Med.

[5] Epstein RM, Hundert EM (2002). Defining and assessing professional competence. JAMA.

[6] Gontijo ED, Alvim C, Megale L, Melo JRC (2013). Essential competences for the training and evaluation of performance in undergraduate medical education. Rev. Bras. Educ. Med.

[7] Pereira GA, Pereira GA, Guedes HTV (2021). Simulação em saúde para ensino e avaliação: conceitos e práticas.

[8] Miller GE (1990). The assessment of clinical skills/competence/performance. Acad Med.

[9] Norcini J, Lippner RJ, Grosso LI (2013). Assessment in the context of licensure and certification. Teach Learn Med.

[10] Shumway JM, Harden RM (2003). AMME Guide No 25: the assessment of learning outcomes for the competent and reflective physician. Med Teach.

[11] Van Der Vleuten C.Schuwirth L.Scheele F.Driessen E.Hodges B (2010). The assessment of professional competence building blocks for theory development. Best Pract Res Clin Obstet Gynaecol.

[12] Wass V, Bowden R, Jackson N, Jackson N, Jamieson A, Khan A (2007). Assessment in medical education and training.

